# Seven new hypselostomatid species from China, including some of the world’s smallest land snails (Gastropoda, Pulmonata, Orthurethra)

**DOI:** 10.3897/zookeys.523.6114

**Published:** 2015-09-28

**Authors:** Barna Páll-Gergely, András Hunyadi, Adrienne Jochum, Takahiro Asami

**Affiliations:** 1 Department of Biology, Shinshu University, Matsumoto 390-8621, Japan; 2 Adria sétány 10G 2/5., Budapest 1148, Hungary; 3 Naturhistorisches Museum der Burgergemeinde Bern, CH-3005 Bern, Switzerland; 4 Institute of Ecology and Evolution, University of Bern, 3012 Bern, Switzerland

**Keywords:** Taxonomy, land snail, dwarfism, Pupillidae, Vertiginidae, apertural barriers

## Abstract

Seven new species of Hypselostomatidae are described from the Chinese province Guangxi: *Angustopila dominikae* Páll-Gergely & Hunyadi, **sp. n.**, *Angustopila fabella* Páll-Gergely & Hunyadi, **sp. n.**, *Angustopila subelevata* Páll-Gergely & Hunyadi, **sp. n.**, *Angustopila szekeresi* Páll-Gergely & Hunyadi, **sp. n.**, *Hypselostoma socialis* Páll-Gergely & Hunyadi, **sp. n.**, *Hypselostoma lacrima* Páll-Gergely & Hunyadi, **sp. n.** and *Krobylos sinensis* Páll-Gergely & Hunyadi, **sp. n.** The latter species is reported from three localities. All other new species are known only from the type locality. Specimens nearly identical to the type specimens of *Angustopila huoyani* Jochum, Slapnik & Páll-Gergely, 2014 were found in a cave in northern Guangxi, 500 km from the type locality. Adult individuals of *Angustopila subelevata* **sp. n.** (shell height = 0.83–0.91 mm, mean = 0.87 mm) and *Angustopila dominikae* **sp. n.** (shell height of the holotype = 0.86 mm) represent the smallest known members of the Hypselostomatidae, and thus are amongst the smallest land snails ever reported. We note that *Pyramidula laosensis* Saurin, 1953 might also belong to *Krobylos*. *Paraboysidia neglecta* van Benthem Jutting, 1961, which was previously included in *Angustopila*, is classified in *Hypselostoma*.

## Introduction

The term “microsnail” usually refers to gastropods with shells smaller than 5 mm (Panha and Burch 2005). Species within this size range do not form a monophyletic unit. Hence, the term “microsnail” is used in the practical sense only. Microgastropods represent a large portion of worldwide and tropical land snail diversity. Knowledge about their biodiversity is scant due to two main reasons: i) many microsnails are reported from caves only or known to inhabit rock outcrops, meaning that they can only be effectively collected using special techniques, such as sieving from soil samples; ii) many microsnails are reported from small ranges and often from the type locality only (e.g. Neubert and Bouchet 2015). However, microsnails can also tend to inhabit the broadest ranges known for land snails (e.g. Vertiginidae, *Carychium*; Nekola and Coles 2010, Weigand et al. 2013, Nekola 2014).

High rates of endemism amongst tropical microsnails requires researchers to perform detailed samplings over large geographic areas in order to find the narrow range endemic species. Superordinate systematics (genus and above) of small-shelled gastropods confronts similar difficulties. Since finding live populations is a challenging endeavour, classification is largely conchologically driven.

One of the families known to contain particularly tiny species is the family Hypselostomatidae, introduced by Zilch (1959) as a subfamily of Chondrinidae. The subfamily Aulacospirinae was also erected in the same work. Schileyko (1998a) synonymized these two taxa because no diagnostic characters were designated by Zilch (1959). The family Hypselostomatidae sensu Schileyko (1998a) inhabits Indochina, Indonesia, Australia and the Philippines, and contains the following genera: *Boysidia* Ancey, 1881 (with the subgenera *Paraboysidia* Pilsbry, 1917 and *Dasypupa* Thompson & Dance, 1983), *Anauchen* Pilsbry, 1917, *Bensonella* Pilsbry & Vanatta, 1900, *Aulacospira* Möllendorff, 1890, *Pseudostreptaxis* Möllendorff, 1890, *Gyliotrachela* Tomlin, 1930, *Hypselostoma* Benson, 1856, *Campolaemus* Pilsbry, 1892, *Boysia* L. Pfeiffer, 1849 and *Acinolaemus* Thompson & Upatham, 1997 (Schileyko 1998a). These genera, together with *Systenostoma* Bavay & Dautzenberg, 1909 are sometimes included in the Pupillidae (e. g. Panha and Burch 1999) or in theVertiginidae (e.g. Thompson and Upatham 1997). Schileyko (1998b) concluded that *Systenostoma* probably does not belong to Hypselostomatidae, but likely belongs to the Helicodiscidae due to the characteristic spiral sculpture. Later, he postulated that the genus is possibly related to *Aulacospira* as considered by Pilsbry (1917) or to *Pupisoma* Stoliczka, 1873 (Valloniidae) (Schileyko 2011). Jochum et al. (2014) renamed *Systenostoma* Bavay & Dautzenberg, 1909 (non *Systenostoma* Marsson, 1887, Bryozoa) as *Tonkinospira* Jochum, Slapnik & Páll-Gergely, 2014, and erected a new genus (*Angustopila* Jochum, Slapnik & Páll-Gergely, 2014) for some species which were previously classified within *Systenostoma*. Jochum et al. (2014) claimed that *Angustopila* probably belongs to the Hypselostomatidae, but the taxonomic position of *Tonkinospira* within the family remained uncertain. We include all genera in question (*Angustopila*, *Hypselostoma*, *Krobylos* Panha & Burch, 1999, *Tonkinospira*) in Hypselostomatidae.

In the present work, seven new species recently collected in Guangxi Province, China are described, belonging to the genera *Angustopila*, *Hypselostoma* and *Krobylos*. We also highlight some difficulties in the pre-existing practice of ranking species into genera based on conchological characters.

## Materials and methods

Shells were first wetted in a dish of water and then manually brushed clean of mud using fine, tapered brushes, whereby each specimen was gently rotated back and forth between the brushes until it was sediment free. The shells were viewed without coating under a low vacuum SEM (Miniscope TM-1000, Hitachi High-Technologies, Tokyo). Shell whorl number was counted to the nearest quarter whorl according to Kerney and Cameron (1979).

Measurements of *Angustopila* and *Hypselostoma* specimens were taken from images obtained by a Nikon Digital Sight DS-FI1 microscope camera attached to a Nikon SMZ 800 Zoom Stereomicroscope. *Krobylos* specimens were measured using digital Vernier callipers. For the species descriptions, shell measurements are expressed as ratios such as SW/SH and AW/AH.

### Abbreviations

HA Collection András Hunyadi (Budapest, Hungary)

HNHM Magyar Természettudományi Múzeum (Budapest, Hungary)

MNHN Muséum National d’Histoire Naturelle (Paris, France)

NMBE Naturhistorisches Museum der Burgergemeinde Bern, (Bern, Switzerland)

NHMUK The Natural History Museum (London, UK)

PGB Collection Barna Páll-Gergely (Mosonmagyaróvár, Hungary)

SMF Senckenberg Forschungsinstitut und Naturmuseum (Frankfurt am Main, Germany)

## Taxonomic descriptions

### Genus Angustopila

Taxon classificationAnimaliaPulmonataHypselostomatidae

Jochum, Slapnik & Páll-Gergely, 2014

Angustopila Jochum, Slapnik & Páll-Gergely, 2014; Jochum et al. 2014: 410: 26.

#### Type species.

*Systenostoma tamlod* Panha & Burch, 1999, by original designation.

#### Including.

*concava* (Thompson & Upatham, 1997), *dominikae* Páll-Gergely & Hunyadi, sp. n., *elevata* (Thompson & Upatham, 1997), *huoyani* Jochum, Slapnik & Páll-Gergely, 2014, *fabella* Páll-Gergely & Hunyadi, sp. n., *subelevata* Páll-Gergely & Hunyadi, sp. n., *szekeresi* Páll-Gergely & Hunyadi, sp. n., *tamlod* (Panha & Burch, 1999).

#### Remarks.

*Paraboysidia neglecta* van Benthem Jutting, 1961 was classified within the genus *Systenostoma* by Panha and Burch and in *Angustopila* by Jochum et al. (2014) due to the presence of only two teeth in the aperture. The wide umbilicus and the detached peristome are, however, very similar to the members of the genus *Hypselostoma* (material examined: Caves near Biserat, state of Jalor, Malay Peninsula, NHMUK 1901.07.19.24–27, syntypes). Therefore we reclassify *Paraboysidia neglecta* in *Hypselostoma*.

### Angustopila dominikae

Taxon classificationAnimaliaPulmonataHypselostomatidae

Páll-Gergely & Hunyadi sp. n.

http://zoobank.org/6C7AF4AA-D0FF-4CB5-BD7F-ADD52654945C

Figure 1 , 12

#### Type material.

China, Guangxi (广西), Hechi Shi (河池市), Bama Xian (巴马县), cliffs at the southern edge of Jiaole Cun (交乐村), 590 m, 24°7.045'N, 107°7.847'E, leg. Hunyadi, A. & Szekeres, M., 10.09.2013., HNHM 99435 (holotype).

#### Diagnosis.

A tiny, corpulent species with elongated aperture having a parietal and a single palatal tooth.

#### Description of the holotype.

Shell minute, light grey, corpulent, almost globular, the penultimate whorl is the widest from apertural view; protoconch consists of 1.5 whorls, protoconch microstructure finely pitted and granular with a powdery superficial texture, the granular microstructure collectively radiates from the nuclear whorl and ceases at the second; teleoconch finely ornamented with irregularly-spaced radial growth lines crossed by fine rows of equidistantly spaced microscopic spiral threads; the 4.75 whorls are separated by a deep suture; whorls shouldered; aperture slightly oblique to shell axis; umbilicus deep, very narrow; aperture elliptical; the sinulus is narrow; peristome slightly expanded, not reflected; the mid section comprising the parietal tooth is sinuous and slightly protruding (in side view); parietal callus well developed, its portion between the parietal tooth and the columella adnate to the penultimate whorl; the portion of the callus between the parietal tooth and the upper right sinulus edge is detached; parietal tooth well developed with a very small additional tubercle (may be homologous with the angular tooth), the palatal tooth is positioned deeper in the shell and directly opposite the parietal tooth.

**Figure 1.**
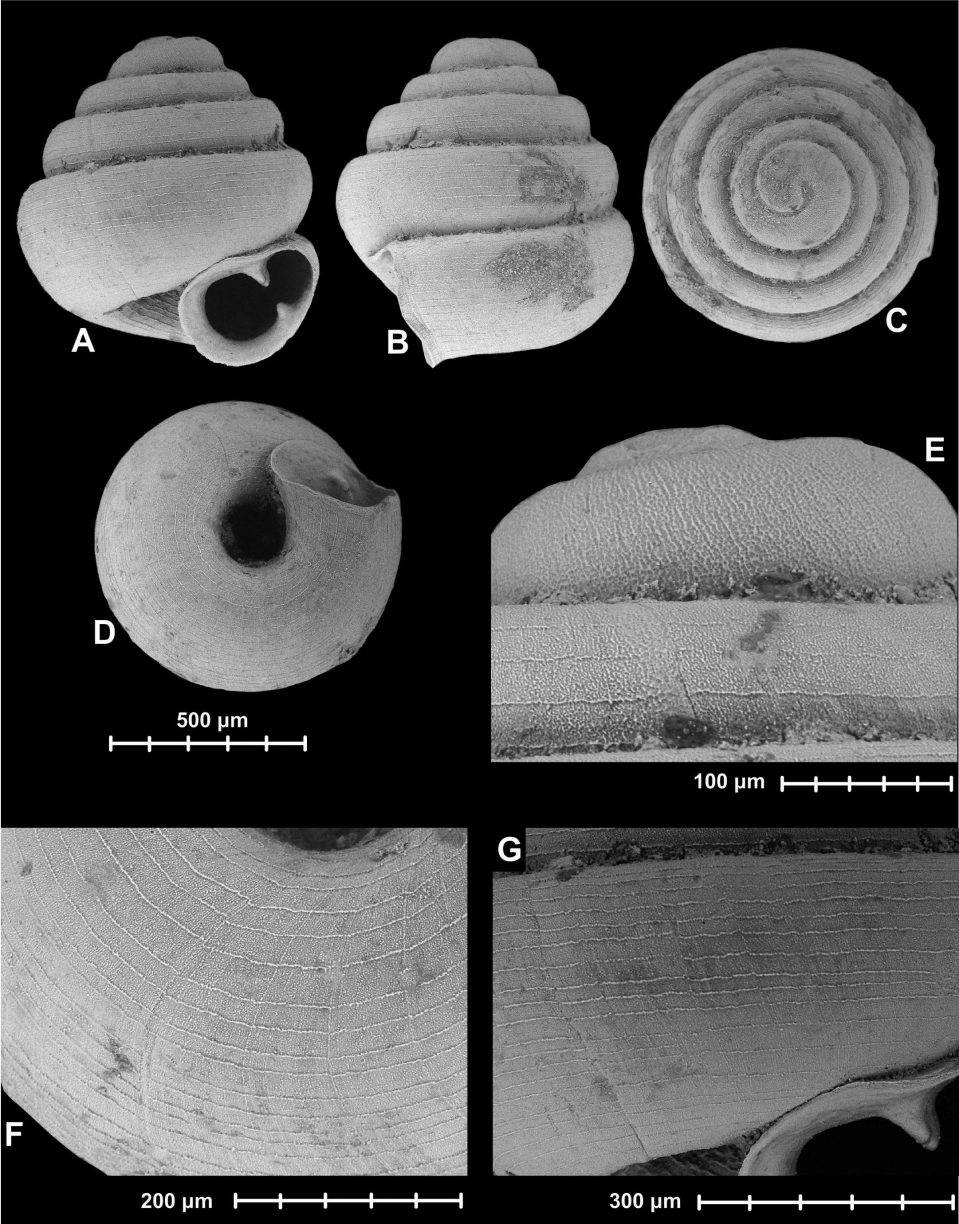
Holotype of *Angustopila dominikae* Páll-Gergely & Hunyadi, sp. n. (HNHM 99435). All images: B. Páll-Gergely.

#### Measurements

(in mm): SH = 0.86, SW = 0.8, AH = 0.3, AW = 0.37, SW/SH×100 = 93.02, AW/AH×100 = 123.33 (n = 1).

#### Differential diagnosis.

*Angustopila tamlod* from Thailand also possesses two teeth (parietal and palatal), but it has a conical shell, which is nearly globular in *Angustopila dominikae* sp. n. Moreover, *Angustopila tamlod* has a narrower umbilicus and a more rounded aperture. *Angustopila huoyani* is larger than *Angustopila dominikae* sp. n. It has a rather conical shell, more whorls, a narrower umbilicus, two apertural denticles and lacks the spiral thread-like lines (or has much weaker spiral striae) on the whole shell. The sympatric *Angustopila subelevata* sp. n. has a conical shell and lacks apertural dentition. See also under *Angustopila fabella* sp. n. and *Angustopila szekeresi* sp. n.

#### Etymology.

The new species is named after Mrs. Dominika Páll-Gergely, the wife of the first author.

#### Type locality.

China, Guangxi (广西), Hechi Shi (河池市), Bama Xian (巴马县), cliffs at the southern edge of Jiaole Cun (交乐村), 590 m, 24°7.045'N, 107°7.847'E.

#### Distribution.

The new species is known from the type locality only (Figure 13).

#### Ecology.

The single empty shell of this new species was found in a soil sample at the base of limestone rocks. It likely lives on limestone walls as do other similar hypselostomatid species recorded by Panha and Burch (2005).

#### Conservation status.

A single empty shell has been collected from a soil sample at the type locality. Therefore, knowledge is very limited for evaluating its conservation status. Since the species is known from one site only, it is evaluated as Critically Endangered (CR) under IUCN criteria (IUCN 2014). Quarrying is quoted as the main threat to similar limestone habitats. However, no ongoing threats to the type locality are known at the moment.

### Angustopila fabella

Taxon classificationAnimaliaPulmonataHypselostomatidae

Páll-Gergely & Hunyadi sp. n.

http://zoobank.org/E5FDAE89-5B6F-419D-BABE-2A10F0144622

Figure 2

#### Type material.

China, Guangxi (广西), Chongzuo Shi (崇左市), Longzhou Xian (龙州县), cliffs north of Lenglei (楞垒), north of the Nonggang Nature Reserve (弄岗国家级自然保护区), 220 m, 22°29.161'N, 106°57.357'E, leg. Hunyadi, A. & Szekeres, M., 23.09.2013., HNHM 99436 (holotype), HNHM 99437/2 (figured paratypes), SMF 346520/1 paratype, HA/38 paratypes + 2 juvenile shells (not paratypes), PGB/1 paratype.

#### Diagnosis.

A tiny, trigonal-shaped species with a rather rounded, slightly bean-shaped aperture bearing a well-developed parietal tooth.

#### Description.

Shell minute, light grey, bluntly trigonal; protoconch consists of slightly more than 1.25 whorls, protoconch microstructure finely pitted and granular with a powdery superficial texture, the granular microstructure collectively radiates from the nuclear whorl and ceases at the second; teleoconch finely reticulate with irregularly-spaced radial growth lines crossed by rows of microscopic spiral threads; the 4.5–4.75 whorls are separated by a deep suture; whorls shouldered; aperture slightly oblique to shell axis; umbilical zone highly reticulate, umbilicus deep, relatively narrow; aperture heart-shaped; peristome slightly expanded, not reflected; parietal callus well-developed, very slightly adnate to the penultimate whorl; parietal tooth prominent, thick and long; no other dentition is present. Body whorl bulges beyond aperture (side view) by ca. 1/7 the max. breadth of the shell. Apertural lip tilted slightly back with fine creases behind the peristome (side view).

**Figure 2.**
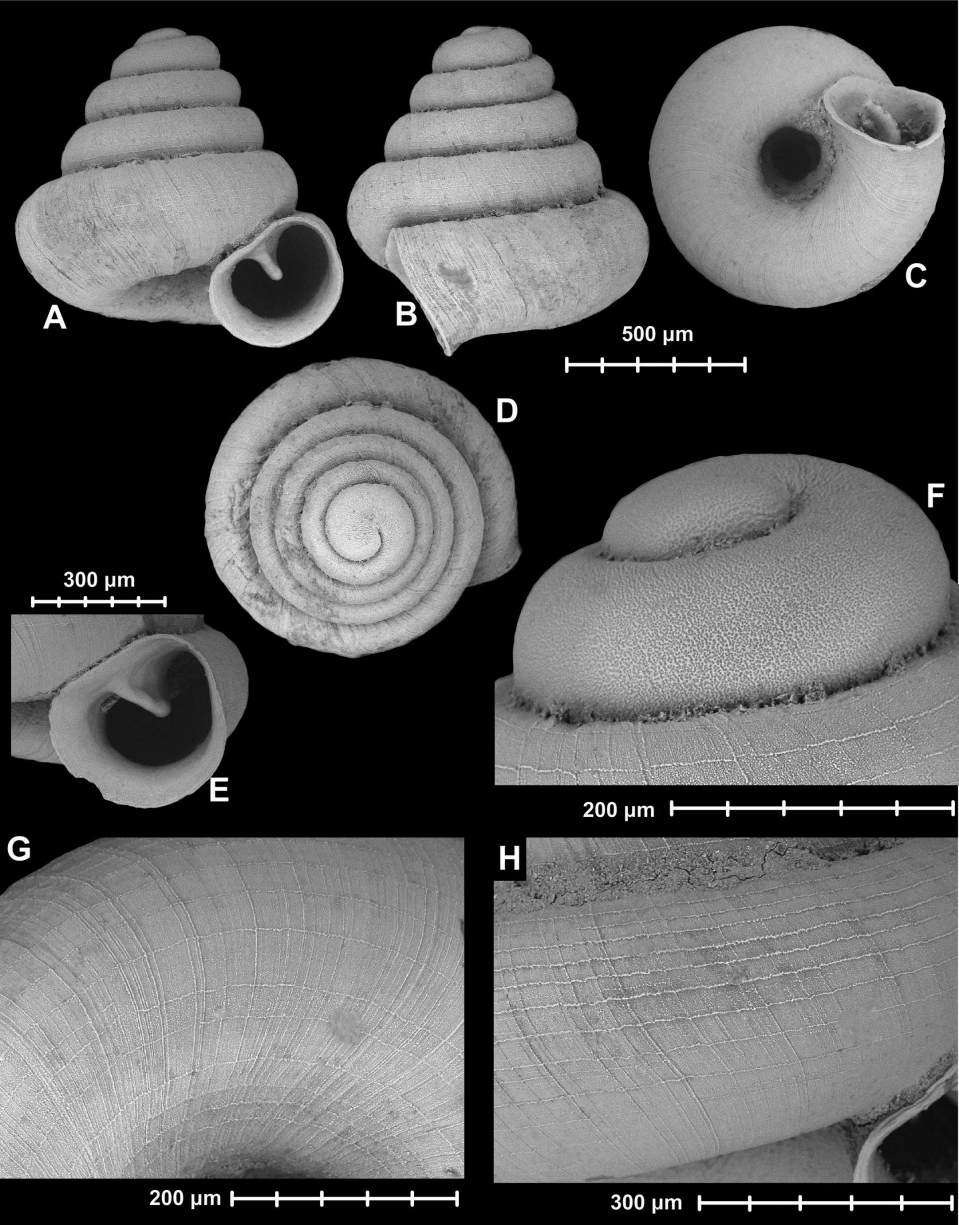
*Angustopila fabella* Páll-Gergely & Hunyadi, sp. n. Holotype: (HNHM 99436: **A, B, D, F**), Paratype1 (HNHM 99437: **E**), Paratype2 (HNHM 99437: **C, G, H**). All images: B. Páll-Gergely.

#### Measurements

(in mm): SH = 0.86–1.02, SW = 0.88–1, AH = 0.34–0.4, AW = 0.36–0.41 (n = 20). See also Tables 1 and 2.

**Table 1.** Shell measurements (mm) for *Angustopila fabella* sp. n. from the type locality. SH: shell height, SW: shell width, AH: aperture height, AW: aperture width, SW/SH×100: shell width shared with shell height and multiplied by 100, AW/AH×100: aperture width shared with aperture height and multiplied by 100.

Specimen	SH	SW	AH	AW	SW/SH×100	AW/AH×100
holotype	0.97	1	0.37	0.4	103.09	108.11
paratype1	0.96	0.98	0.39	0.41	102.08	105.13
paratype2	0.96	0.92	0.37	0.38	95.83	102.7
paratype3	1.01	0.94	0.37	0.38	93.07	102.7
paratype4	0.92	0.94	0.36	0.39	102.17	108.33
paratype5	0.86	0.98	0.4	0.4	113.95	100
paratype6	0.93	0.94	0.38	0.39	101.08	102.63
paratype7	0.97	0.93	0.39	0.39	95.88	100
paratype8	0.96	0.94	0.39	0.39	97.92	100
paratype9	0.99	0.89	0.36	0.39	89.9	108.33
paratype10	1.02	0.94	0.4	0.38	92.16	95
paratype11	0.92	0.93	0.37	0.4	101.09	108
paratype12	0.97	0.94	0.37	0.38	96.91	102.7
paratype13	0.97	0.93	0.37	0.4	95.88	108.11
paratype14	0.94	0.91	0.36	0.38	96.81	105.56
paratype15	0.93	0.88	0.34	0.37	94.61	108.82
paratype16	0.95	0.95	0.39	0.39	100	100
paratype17	0.89	0.89	0.35	0.36	100	102.86
paratype18	0.95	0.93	0.38	0.38	97.89	100
paratype19	0.93	0.91	0.37	0.39	97.85	105.41

**Table 2.** Average, minimum value (min), maximum value (max), variance of values (var) and standard deviation of a set of values (stdev) for *Angustopila fabella* sp. n. (n = 20).

	SH	SW	AH	AW	SW/SH×100	AW/AH×100
Average	0.95	0.9335	0.374	0.3875	98.4085	103.7195
Min	0.86	0.88	0.34	0.36	89.9	95
Max	1.02	1	0.4	0.41	113.95	108.82
Var	0.0014	0.0009	0.0003	0.0001	25.7841	14.9414
stdev	0.0376	0.0301	0.016	0.0116	5.0778	3.8654

#### Differential diagnosis.

*Angustopila fabella* sp. n. is most similar to *Angustopila tamlod* in shape and form. However, in addition to the parietal denticle, *Angustopila tamlod* has a small, low palatal plica just opposite the parietal denticle. *Angustopila dominikae* sp. n. is smaller, has a globular shell (conical in *Angustopila fabella* sp. n.) and possesses two apertural denticles with an additional tubercle on the parietal denticle. A single parietal denticle is present in *Angustopila fabella* sp. n. See also *Angustopila subelevata* sp. n. and *Angustopila szekeresi* sp. n.

#### Etymology.

The name, fabella, (Latin: little bean) refers to the bean-shaped aperture.

#### Type locality.

China, Guangxi (广西), Chongzuo Shi (崇左市), Longzhou Xian (龙州县), cliffs north of Lenglei (楞垒), north of the Nonggang Nature Reserve (弄岗国家级自然保护区), 220 m, 22°29.161'N, 106°57.357'E.

#### Distribution.

*Angustopila fabella* sp. n. is known from the type locality only (Figure 13).

#### Ecology.

Empty shells of this new species were found in a soil sample at the base of large limestone rocks. It likely lives on limestone walls as do other similar hypselostomatid species recorded by Panha and Burch (2005).

#### Conservation status.

Empty shells have been collected from a soil sample at the type locality. Therefore, knowledge is very limited for evaluating its conservation status. Since the species is known from one site only, it is evaluated as Critically Endangered (CR) under IUCN criteria (IUCN 2014). Quarrying is quoted as the main threat to similar limestone habitats. However, no ongoing threats to the type locality are known at the moment.

### Angustopila huoyani

Taxon classificationAnimaliaPulmonataHypselostomatidae

Jochum, Slapnik & Páll-Gergely, 2014

Figure 3

Angustopila huoyani Jochum, Slapnik & Páll-Gergely, 2014: Jochum et al. 2014: 410: 27–29, Video 1, Figs 4–5.

#### Material examined.

MNHN Expedition Nr. GX07.23.07, China, Guangxi (广西), Hechi (河池市), Huanjiang Xian (环江县), Midong village (米洞), Shui Dong (cave, 水洞), 23.05.2007, river sediment, alt. 332 m, 24.7485°N, 108.27191°E, leg. Franck Bréhier 12 shells (2 broken), NMBE 535121/3, SMF 341637/3, MNHN 2012-27046/4 + 2 broken shells).

#### Conservation status.

This study reveals that *Angustopila huoyani* inhabits two caves that are geographically far from each other. The typical threats to such habitats is quarrying and human disturbance through tourism.

#### Remarks.

*Angustopila huoyani* has been described from a single cave in northeastern Hunan Province. Nearly identical shells have been found in another cave in northern Guangxi Province, which is situated ca. 500 km south from the type locality. The only difference is that the new shells have some very faint spiral striae on the teleoconch, which were not detected in the original population. This difference is, however, insufficient to distinguish these two populations on either specific or subspecific level. Therefore, we refer to the population collected in Guangxi as a disjunct population of *Angustopila huoyani*. This finding underscores the need to explore more cave systems in order to make inferences about subterranean biodiversity in China, and specifically here for the distribution of minute troglobitic land snails.

**Figure 3.**
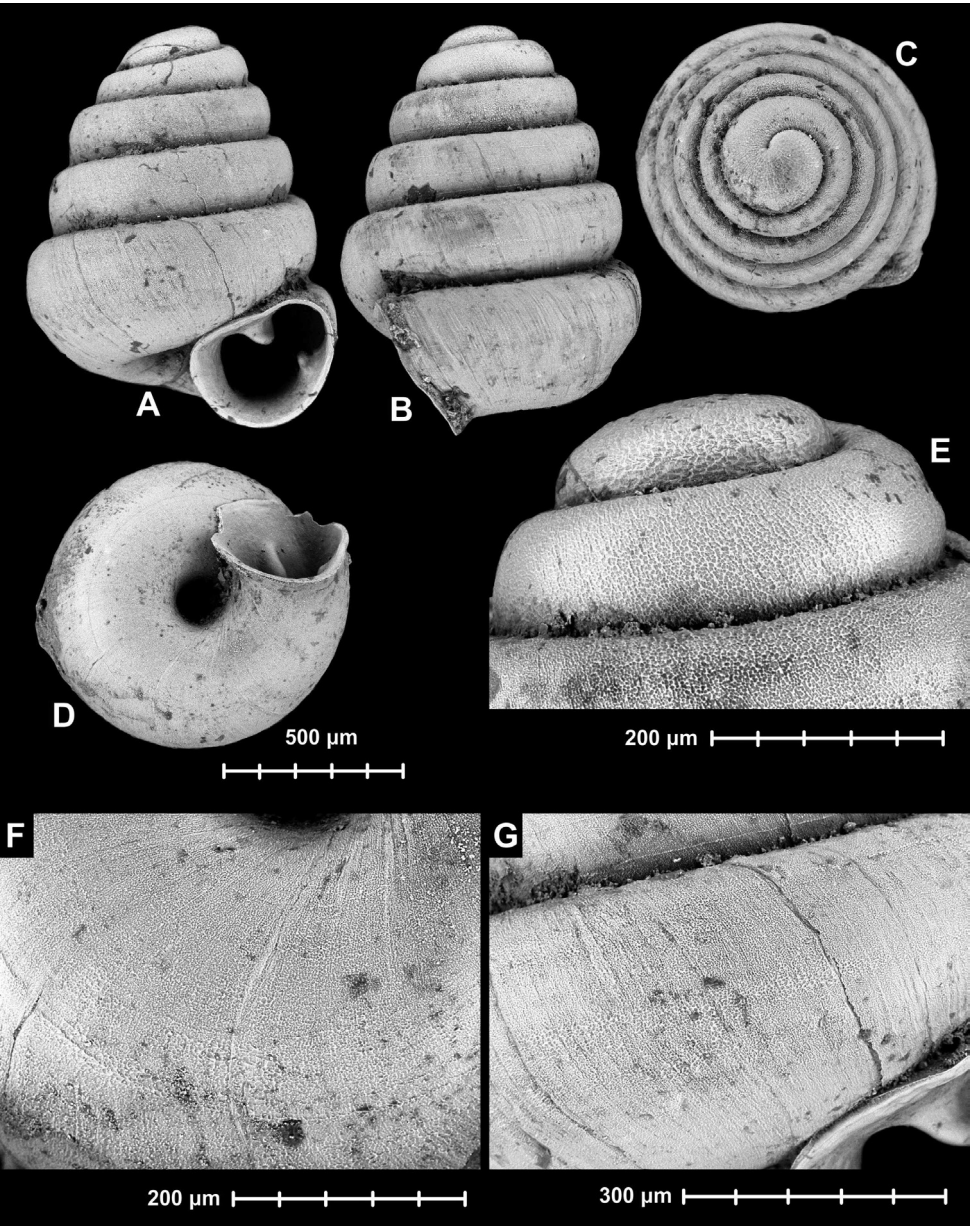
*Angustopila huoyani* Jochum, Slapnik & Páll-Gergely, 2014. Locality: Guangxi (广西), Hechi (河池市), Huanjiang Xian (环江县), Midong village (米洞), Shui Dong (cave, 水洞), 23.05.2007, river sediment, alt. 332 m, 24.7485°N, 108.27191°E. MNHN 2012-27046). All images: B. Páll-Gergely.

### Angustopila subelevata

Taxon classificationAnimaliaPulmonataHypselostomatidae

Páll-Gergely & Hunyadi sp. n.

http://zoobank.org/74DACAA7-B195-459F-B39E-B11D875FD015

Figure 4

#### Type material.

China, Guangxi (广西), Hechi Shi (河池市), Bama Xian (巴马县), cliffs at the southern edge of Jiaole Cun (交乐村), 590 m, 24°7.045'N, 107°7.847'E, leg. Hunyadi, A. & Szekeres, M., 10.09.2013., HNHM 99438 (holotype), HNHM 99439/1 (paratype), HA/10 paratypes.

#### Diagnosis.

A tiny, conical species with rounded or almost quadrangular aperture without dentition.

#### Description.

Shell minute, light grey, conical with obtuse apex; spire tilted slightly left; protoconch consists of 1.25–1.5 whorls, microstructure finely pitted and granular with a powdery superficial texture, collectively radiating from the nuclear whorl; a prominent protoconch/teleoconch boundary is present (p/t), which is preceded by very faint rows of finely threaded microstructure; teleoconch finely reticulate with regularly-spaced radial growth lines crossed by rows of microscopic spiral threads.; on the last whorl, every 5^th^–6^th^ radial line is stronger; the 4.25 whorls are separated by a deep suture; whorls shouldered; body whorl tumid; aperture slightly oblique to shell axis; umbilicus deep, relatively wide; aperture rounded or almost quadrangular, toothless; peristome slightly expanded, not reflected; parietal margin extends forward as a slight tongue-like projection along the columellar curvature; outer lip (side view) arched slightly and drawn back below suture.

**Figure 4.**
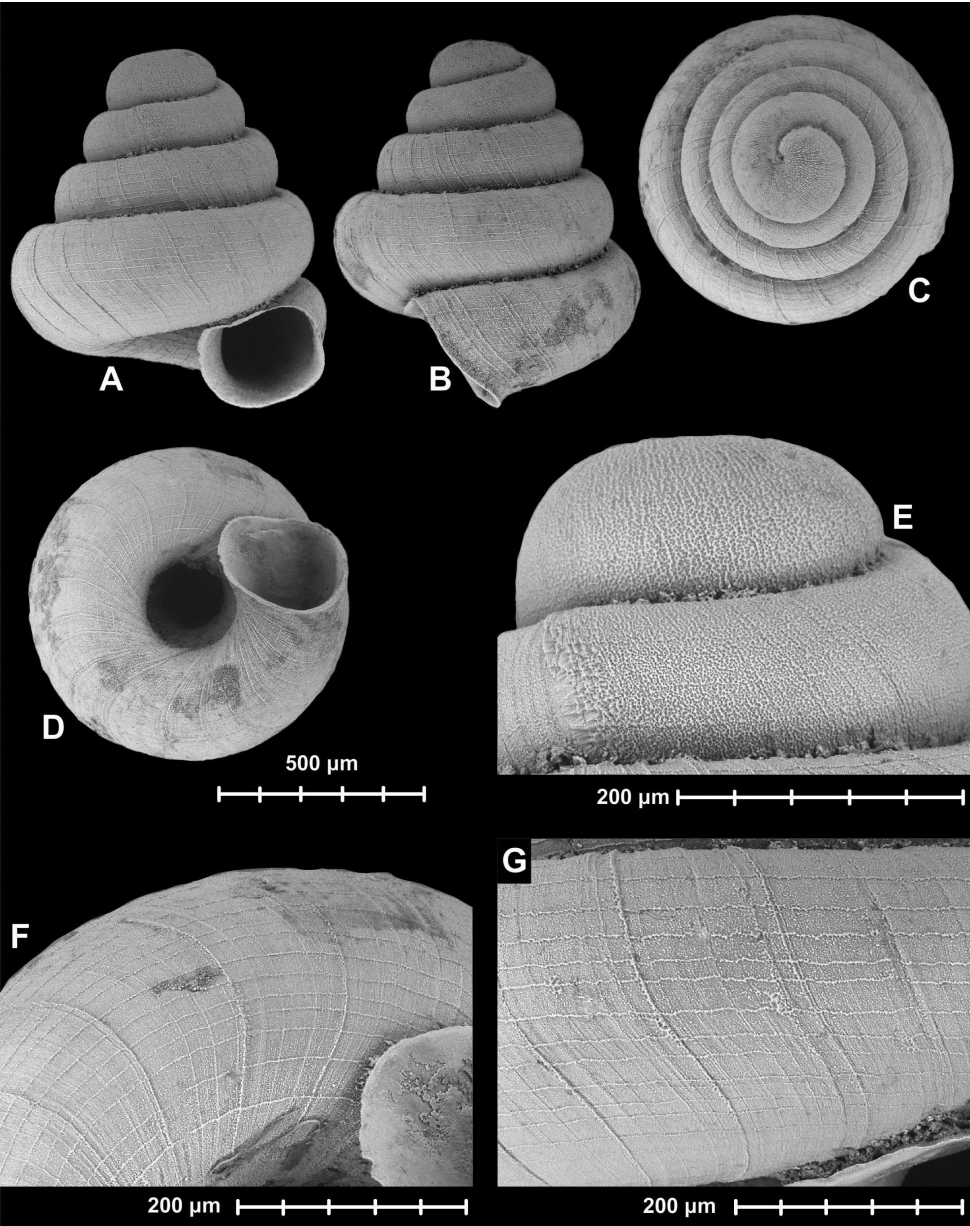
Holotype of *Angustopila subelevata* Páll-Gergely & Hunyadi, sp. n. (HNHM 99438). All images: B. Páll-Gergely.

#### Measurements

(in mm): SH = 0.83–0.91, SW = 0.77–0.81, AH = 0.27–0.3, AW = 0.29–0.32 (n = 8). See also Tables 3 and 4.

**Table 3.** Shell measurements (mm) for *Angustopila subelevata* sp. n. from the type locality. For abbreviations see Table 1.

Specimen	SH	SW	AH	AW	SW/SH×100	AW/AH×100
holotype	0.88	0.8	0.3	0.31	90.91	103.33
paratype1	0.87	0.81	0.29	0.32	93.1	110.34
paratype2	0.86	0.77	0.3	0.32	89.53	106.67
paratype3	0.88	0.79	0.28	0.29	89.77	103.57
paratype4	0.85	0.78	0.3	0.32	91.76	106.67
paratype5	0.91	0.79	0.27	0.31	86.81	114.81
paratype6	0.86	0.79	0.3	0.31	91.86	103.33
paratype7	0.83	0.81	0.3	0.3	97.59	100

**Table 4.** Average, minimum value (min), maximum value (max), variance of values (var) and standard deviation of a set of values (stdev) for *Angustopila subelevata* sp. n. (n= 8). For abbreviations see Table 1.

	SH	SW	AH	AW	SW/SH×100	AW/AH×100
Average	0.8675	0.7925	0.2925	0.31	91.4163	106.09
Min	0.83	0.77	0.27	0.29	86.81	100
Max	0.91	0.81	0.3	0.32	97.59	114.81
Var	0.0006	0.0002	0.0001	0.0001	9.8582	21.9211
stdev	0.0238	0.0139	0.0116	0.0107	3.1398	4.682

#### Differential diagnosis.

The most similar species is the Thai *Angustopila elevata*, which has a more slender shell, a deeper umbilicus and lacks the spiral striae on its base. *Angustopila fabella* sp. n. has a wider shell, a stronger peristome and a well-developed parietal tooth, whereas *Angustopila subelevata* sp. n. is toothless. See also the two sympatric species, *Angustopila dominikae* sp. n. and *Angustopila szekeresi* sp. n.

#### Etymology.

The name, subelevata, refers to the similarity to the Thai *Angustopila elevata*.

#### Type locality.

China, Guangxi (广西), Hechi Shi (河池市), Bama Xian (巴马县), cliffs at the southern edge of Jiaole Cun (交乐村), 590 m, 24°7.045'N, 107°7.847'E.

#### Distribution.

The new species is known from the type locality only (Figure 13).

#### Ecology.

As for *Angustopila fabella* sp. n.

#### Conservation status.

As for *Angustopila fabella* sp. n.

#### Remarks.

*Angustopila elevata*, which is known from approx. 1,000 km from the type locality of *Angustopila subelevata* sp. n., is strikingly similar to the new species, although the general shell shape and the sculpture seem to be reliably different. See also Discussion.

### Angustopila szekeresi

Taxon classificationAnimaliaPulmonataHypselostomatidae

Páll-Gergely & Hunyadi sp. n.

http://zoobank.org/D9845392-BD63-4253-89F5-B1F89FC779A8

Figure 5

#### Type material.

China, Guangxi (广西), Hechi Shi (河池市), Bama Xian (巴马县), cliffs at the southern edge of Jiaole Cun (交乐村), 590 m, 24°7.045'N, 107°7.847'E, leg. Hunyadi, A. & Szekeres, M., 10.09.2013., HNHM 99440 (holotype), HNHM 99441/2 (one of them is a figured paratype), HA/6 paratypes.

#### Diagnosis.

A tiny, trigonal species with rounded aperture having a weak parietal tooth.

#### Description.

Shell minute, light grey, blunt trigonal; protoconch consists of 1.25 whorls, microstructure finely pitted and granular with a powdery superficial texture, collectively radiating from the nuclear whorl; spiral threads of microstructure transverse the protoconch as well as the teleoconch, a prominent protoconch/teleoconch boundary is present (p/t), which interrupts the very faint rows of finely threaded microstructure; teleoconch finely reticulate with regularly-spaced radial growth striations crossed by rows of microscopic spiral threads; every 8^th^–10^th^ radial line is stronger and visible as growth ridges; the 4–4.25 whorls are separated by a deep suture; whorls rounded; aperture oblique to shell axis; umbilicus deep, relatively narrow; aperture rounded; peristome slightly expanded, not reflected; laterally viewed, the middle section is slightly protruding; parietal callus weak, adnate; parietal tooth weak but present in all specimens.

**Figure 5.**
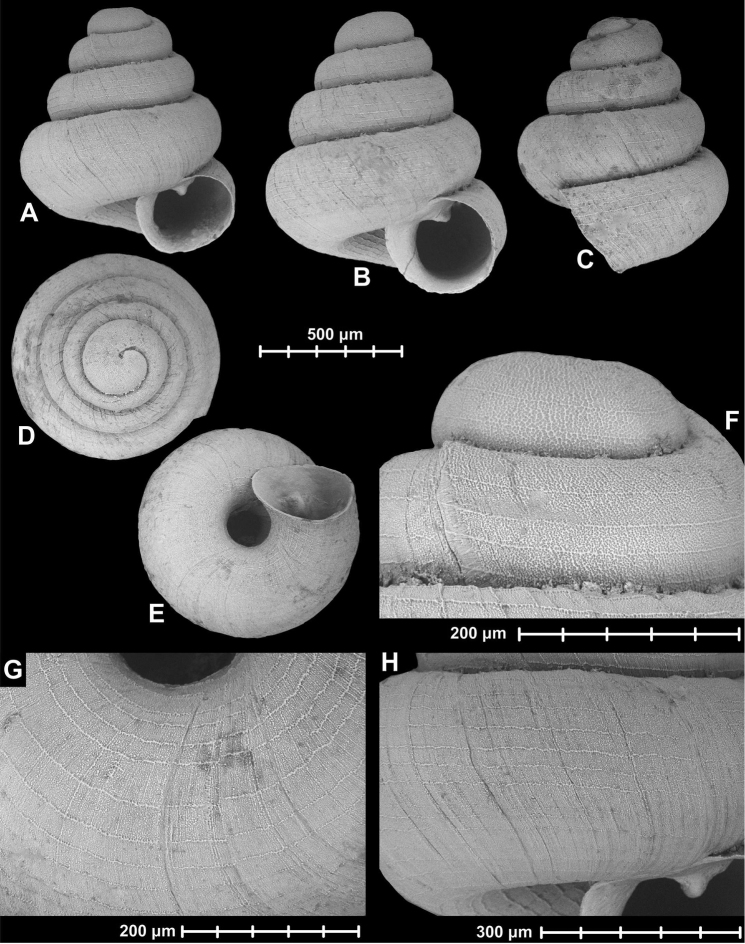
*Angustopila szekeresi* Páll-Gergely & Hunyadi, sp. n. Holotype (HNHM 99440: **A, C, D, E, F, G, H**), Paratype (HNHM 99441: **B**). All images: B. Páll-Gergely.

#### Measurements

(in mm): SH = 0.88–1.03, SW = 0.77–0.89, AH = 0.33–0.37, AW = 0.35–0.39 (n = 6). See also Tables 5 and 6.

**Table 5.** Shell measurements (mm) for *Angustopila szekeresi* sp. n. from the type locality. For abbreviations see Table 1.

Specimen	SH	SW	AH	AW	SW/SH×100	AW/AH×100
Holotype	0.91	0.8	0.34	0.36	87.91	105.88
paratype1	0.93	0.77	0.33	0.35	82.8	106.06
paratype2	1.03	0.89	0.36	0.39	86.41	108.33
paratype3	0.88	0.81	0.37	0.35	92.05	94.59
paratype4	1.03	0.85	0.36	0.39	82.52	108.33
paratype5	0.95	0.8	0.34	0.36	84.21	105.88

**Table 6.** Average, minimum value (min), maximum value (max), variance of values (var) and standard deviation of a set of values (stdev) for *Angustopila szekeresi* sp. n. (n = 6). For abbreviations see Table 1.

	SH	SW	AH	AW	SW/SH×100	AW/AH×100
Average	0.955	0.82	0.35	0.3667	85.9833	104.845
Min	0.88	0.77	0.33	0.35	82.52	94.59
Max	1.03	0.89	0.37	0.39	92.05	108.33
Var	0.0039	0.0018	0.0002	0.0003	13.1943	26.6148
stdev	0.0625	0.0429	0.0155	0.0186	3.6324	5.159

#### Differential diagnosis.

***Sympatric species.*** *Angustopila subelevata* sp. n. lacks a parietal tooth, it has a wider umbilicus, a smaller aperture, and its peristome is not adnate. Moreover, the spiral lines on the embryonic whorls are much weaker in *Angustopila subelevata* sp. n. *Angustopila dominikae* sp. n. is smaller, has a much more corpulent shell and two teeth in the aperture. *Hypselostoma socialis* sp. n. is much larger and has four teeth in its aperture.

***Non-sympatric species.*** *Angustopila fabella* sp. n. has a wider shell, a wider umbilicus, weaker spiral lines on its umbilicus, a stronger parietal tooth and a strong parietal callus (its peristome is not adnate).

#### Etymology.

*Angustopila szekeresi* sp. n. is named after Miklós Szekeres, our friend and partner in the field work resulting in all new species reported in this paper.

#### Type locality.

China, Guangxi (广西), Hechi Shi (河池市), Bama Xian (巴马县), cliffs at the southern edge of Jiaole Cun (交乐村), 590 m, 24°7.045'N, 107°7.847'E.

#### Distribution.

The new species is known from the type locality only (Figure 13).

#### Ecology.

As for *Angustopila fabella* sp. n.

#### Conservation status.

As for *Angustopila fabella* sp. n.

#### Remarks.

The spiral threading on the protoconch is common in the Hypselostomatidae (Panha and Burch 2005). Noteworthy, is the transition with the p/t boundary in that the microstructure continues in sync with the subsequent whorls. Normally, this phase of ontogenetic development in gastropods [p/t boundary] indicates the transition from the protoconch embryonal stage, whereby the shell structure changes and continues in the teleoconch constructional phase. The continuous protoconch-teleoconch microstructural condition here suggests likely progenesis in these snails.

### Genus Hypselostoma

Taxon classificationAnimaliaPulmonataHypselostomatidae

Benson, 1856

Hypselostoma Benson, 1856b; The Annals and Magazine of Natural History, ser. 2, no. 17: 342. (nomen novum pro *Tanystoma*Benson 1856a, non Motschulsky, 1845, Carabidae, Coleoptera).

#### Type species.

*Tanystoma tubiferum* Benson, 1856a, by monotypy.

#### Remarks.

See under the genus *Angustopila*.

### Hypselostoma lacrima

Taxon classificationAnimaliaPulmonataHypselostomatidae

Páll-Gergely & Hunyadi sp. n.

http://zoobank.org/F2872829-97AF-49E6-B3FC-CB787EBF8F10

Figures 6 , 8F–K

#### Type material.

China, Guangxi (广西), Chongzuo Shi (崇左市), Longzhou Xian (龙州县), cliffs N of Lenglei (楞垒), N of the Nonggang Nature Reserve (弄岗国家级自然保护区), 220 m, 22°29.161'N, 106°57.357'E, leg. Hunyadi, A. & Szekeres, M., 23.09.2013., HNHM 99444 (holotype), HNHM 99445 (figured paratype), HA/2 paratypes.

#### Diagnosis.

Shell conical, with tumid body whorl and deep umbilicus; aperture with sinulus vertically oriented; tubus detached; aperture with one parietal lamella, one columellar and two palatal teeth; parietal lamella long and nearly straight.

#### Description.

Shell minute, whitish/light grey, conical with enlarged body whorl; protoconch consists of 1.5 or slightly less whorls, finely granulated, with at least six fine spiral striations; teleoconch reticulated and regularly spirally striated with strong, irregular radial lines; the 5.5 or slightly less whorls are separated by a deep suture; whorls sloping and rounded; aperture oblique to shell axis; base of shell broadly umbilicate due to lateral expansion of last whorl; aperture detached from the penultimate whorl; aperture with sinulus vertically oriented (from apertural view); peristome expanded, not reflected, with relatively sharp edge; four apertural barriers; only the angulo-parietal lamella reaches the peristome; angulo-parietal lamella very long and high, not interrupted; it is lowest near the peristome; its posterior (inner) end is not visible in frontal view; its anterior end (closest to the peristome) is bent toward the upper palatal plica, and its posterior end is bent toward the lower palatal plica; columellar and upper palatal folds elevated but short; the posterior end of the upper palatal fold curls toward the lower palatal fold; the lower palatal fold is also well-developed, and shorter than the others.

**Figure 6.**
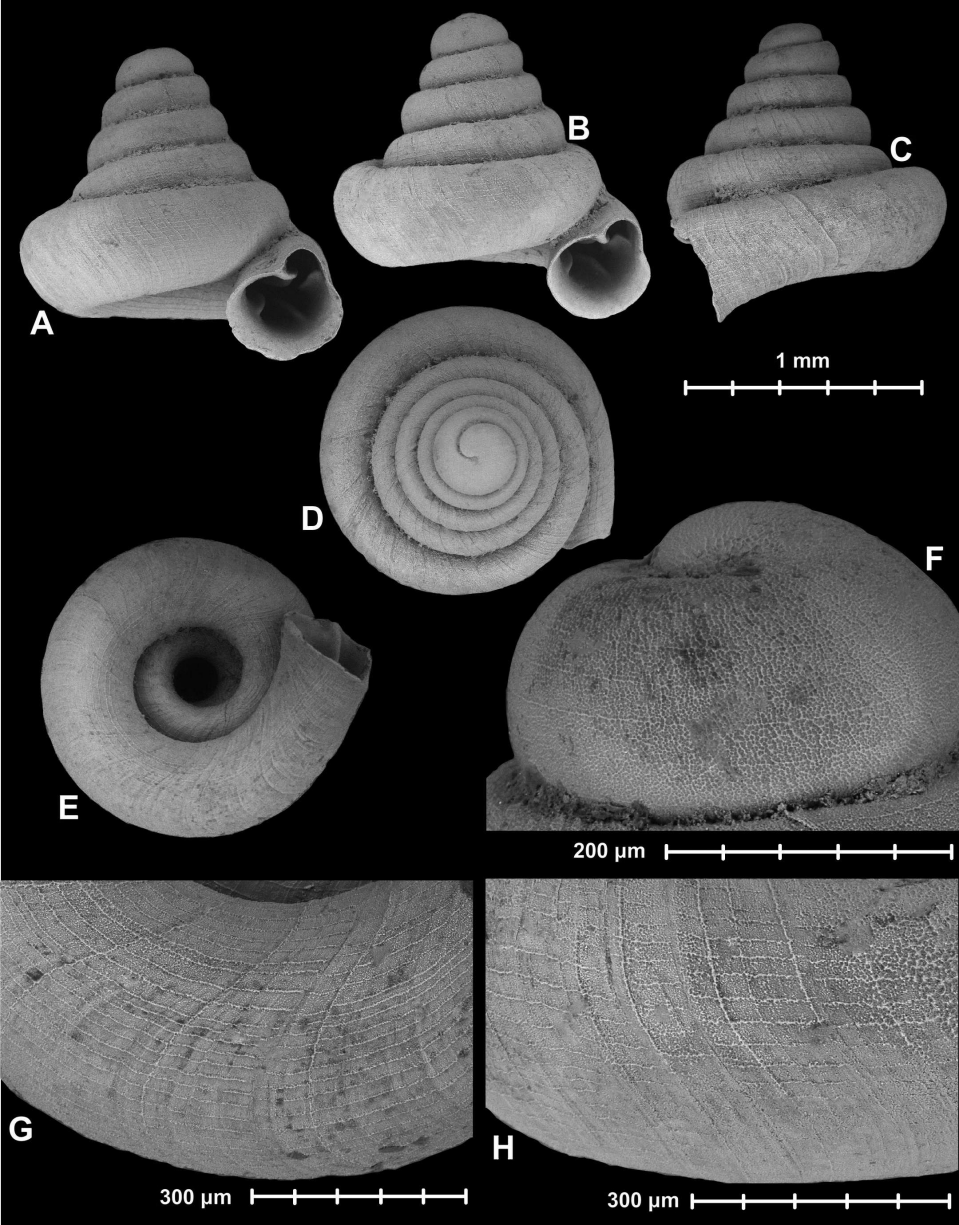
*Hypselostoma lacrima* Páll-Gergely & Hunyadi, sp. n. Holotype (HNHM 99442: **A, C–H**), Paratype (HNHM 99445: **B**). All images: B. Páll-Gergely.

#### Measurements

(in mm): SH = 1.33–1.35, SW = 1.34–1.35, AH = 0.45–0.51, AW = 0.44–0.5 (n = 2). See also Tables 7 and 8.

**Table 7.** Shell measurements (mm) for *Hypselostoma lacrima* sp. n. from the type locality. For abbreviations see Table 1.

Specimen	SH	SW	AH	AW	SW/SH×100	AW/AH×100
holotype	1.33	1.35	0.45	0.44	101.5	125.71
paratype	1.35	1.34	0.51	0.5	99.26	98.04

**Table 8.** Average, minimum value (min), maximum value (max), variance of values (var) and standard deviation of a set of values (stdev) for *Hypselostoma lacrima* sp. n. (n = 2). For abbreviations see Table 1.

	SH	SW	AH	AW	SW/SH×100	AW/AH×100
Average	1.34	1.345	0.48	0.47	100.38	111.875
Min	1.33	1.34	0.45	0.44	99.26	98.04
Max	1.35	1.35	0.51	0.5	101.5	125.71
Var	0.0002	0.0001	0.0018	0.0018	2.5088	382.8144
stdev	0.0141	0.0071	0.0424	0.0424	1.5839	19.5656

#### Differential diagnosis.

See under *Hypselostoma socialis* sp. n.

#### Etymology.

The name lacrima (Latin: tear) refers to the shape of the aperture.

#### Type locality.

China, Guangxi (广西), Chongzuo Shi (崇左市), Longzhou Xian (龙州县), cliffs N of Lenglei (楞垒), N of the Nonggang Nature Reserve (弄岗国家级自然保护区), 220 m, 22°29.161'N, 106°57.357'E.

#### Distribution.

The new species is known from the type locality only (Figure 13).

#### Ecology.

As for *Angustopila fabella* sp. n.

#### Conservation status.

As for *Angustopila fabella* sp. n.

#### Remarks.

The subdivision of Hypselostomatidae is strongly based on the morphology of the apertural barriers (“teeth”). The main characters used for delimiting some of the major genera include the formation of the two teeth on the parietal region of the aperture, namely the parietal tooth (lamella) or parietalis and the angular tooth (lamella) or angularis. *Gyliotrachela*, *Paraboysidia* and *Acinolaemus* are said to possess separate parietal and angular lamellae. The former two have a more prominent parietal lamella rather than angular lamella, but in *Acinolaemus*, the angular is the dominant tooth. The angular lamella is entirely missing in the genus *Anauchen*. In the genera *Hypselostoma* and *Boysidia* these two lamellae are fused (Pilsbry 1917, Thompson and Upatham 1997, Panha and Burch 2005). Sometimes it is challenging to ascertain whether we are dealing with a single lamella (homologous with the parietal lamella) having a bifid anterior end or two lamellae (parietal and angular), which are concrescent. Moreover, the genera *Hypselostoma* and *Gyliotrachela* did not form monophyletic units in the molecular phylogeny presented by Tongkerd et al. (2004), suggesting that the key characters used in classic taxonomy have developed phenotypically plastic forms. In this case of the two new species (*Hypselostoma lacrima* sp. n. and *Hypselostoma socialis* sp. n.), we interpret the lamella on the parietal apertural wall as a congruent angulo-parietal lamella. Hence, both species are placed in *Hypselostoma*.

### Hypselostoma socialis

Taxon classificationAnimaliaPulmonataHypselostomatidae

Páll-Gergely & Hunyadi sp. n.

http://zoobank.org/49F4FD5C-C1E9-4B34-970C-C9B62072329D

Figures 7 , 8A–E

#### Type material.

China, Guangxi (广西), Hechi Shi (河池市), Bama Xian (巴马县), cliffs at the southern edge of Jiaole Cun (交乐村), 590 m, 24°7.045'N, 107°7.847'E, leg. Hunyadi, A. & Szekeres, M., 10.09.2013., HNHM 99442 (holotype), HNHM 99443/3 (figured paratypes), SMF 346521/1 paratype, HA/15 paratypes + 4 juvenile shells (not paratypes), PGB/1.

#### Diagnosis.

Shell spire conical, shell turban-shaped with tumid body whorl and broadly set, deep umbilicus; tubus detached; aperture rounded with wide sinulus, the upper parietal lamella dips to the right; aperture with a parietal lamella, one columellar and two palatal teeth; parietal lamella long and depressed, Z-shaped.

#### Description.

Shell minute, whitish/light grey, conical with enlarged body whorl; protoconch consists of 1.5 whorls, finely pitted, with very slight indication of spiral lines; teleoconch reticulated with fine, regularly spirally striate microstructure intersected with irregular radial lines; the 5.5 whorls are separated by deep suture; whorls horizontally positioned, rounded; aperture oblique to shell axis; umbilicus deep, wide, especially at the last whorl; aperture free from the penultimate whorl, rounded with wide sinulus (area isolated by the parietal and upper palatal lamellae); sinulus horizontally oriented (apertural view); peristome slightly expanded, not reflected, with relatively sharp edge; (side view), the horizontally directed tuba is deflected downwards in alignment with the body whorl; four teeth recessed within aperture; only the ridge-like angulo-parietal lamella reaches the peristome, the others are situated deeper; angulo-parietal lamella moderately long, its end is visible from a straight view into the aperture; it is interrupted, consisting of an anterior section (situated closer to the peristome) and a slightly longer posterior section (situated deeper in the aperture); the anterior section is strongly bent toward the sinulus, its tip nearly touches the tip of the upper palatal fold; the posterior part of the angulo-parietal lamella is less strongly bent than the anterior portion, only its anterior part is bent toward the upper palatal lamella; the angulo-parietal and the upper palatal lamellae follow each other; the angulo-parietal lamella has a depressed Z-shape when observed after breaking off the lower part of the aperture; the anterior part of the angulo-parietal lamella is possibly homologous with the parietal lamella of other hypselostomatid taxa, while the second portion might be homologous with the angular lamella, or vice versa; columellar and lower palatal lamellae are elevated, blunt and short, they are about the same length and are visible through the semi-transparent shell; the upper palatal fold is also of similar length, its posterior end runs parallel with the lower palatal fold; the tip of the upper palatal fold nearly touches the tip of the angulo-parietal lamella.

**Figure 7.**
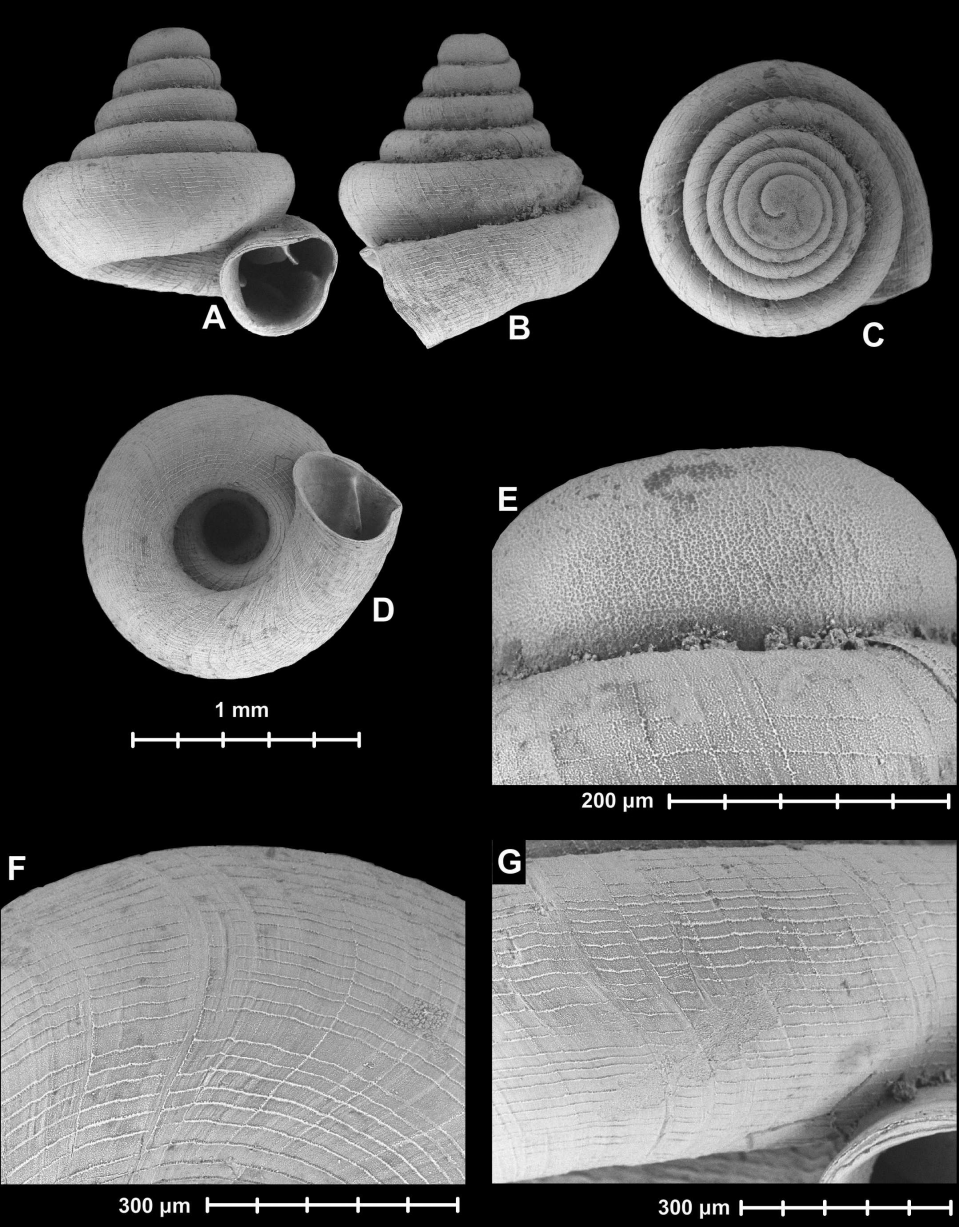
Holotype of *Hypselostoma socialis* Páll-Gergely & Hunyadi, sp. n. (HNHM 99442). All images: B. Páll-Gergely.

**Figure 8.**
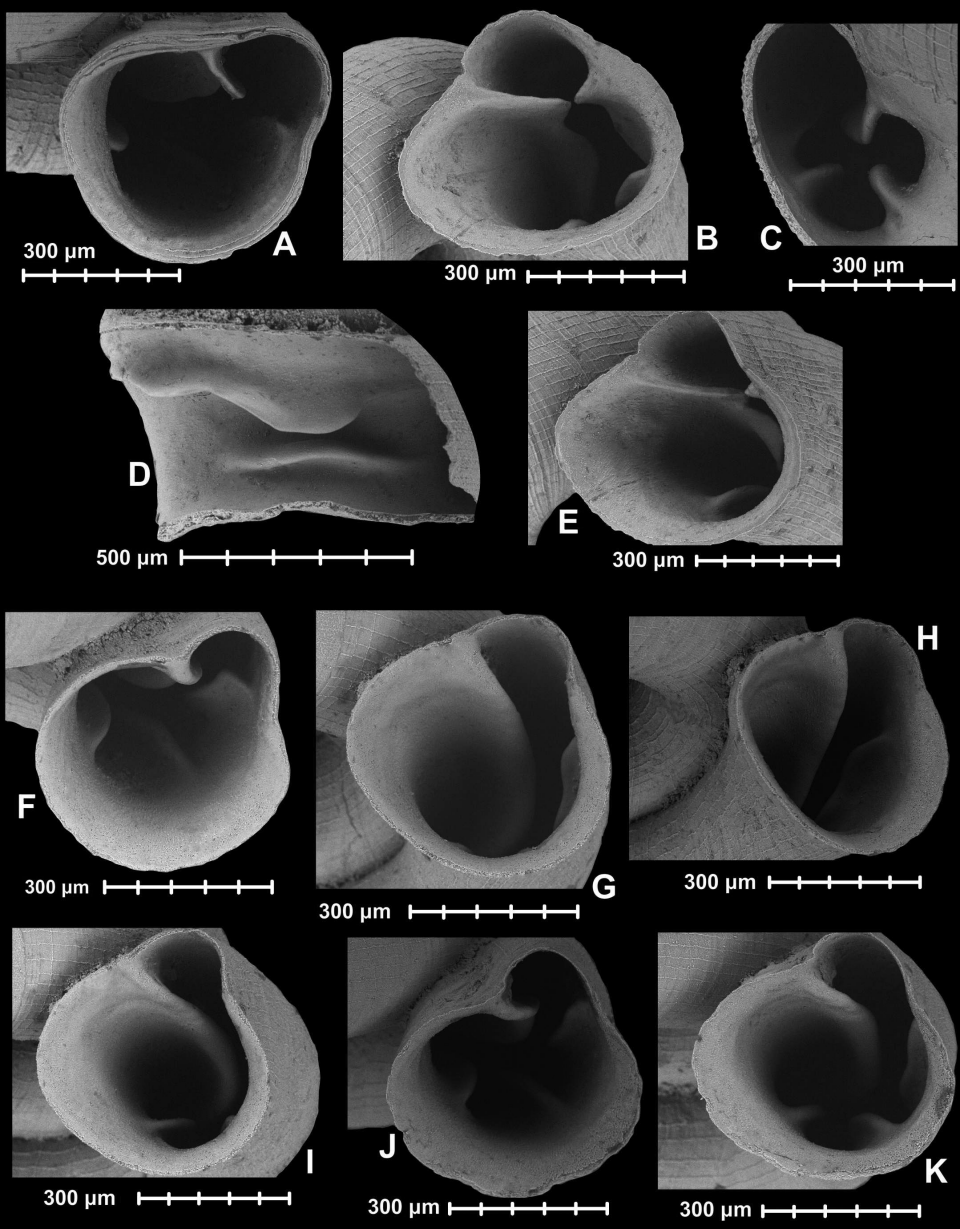
Aperture and apertural barriers of *Hypselostoma* species. **A–E** *Hypselostoma socialis* sp. n.: Holotype (HNHM 99442: **A**) Paratype1 (HNHM 99443: **B, E**), Paratype2 (HNHM 99443: **C**), Paratype3 (HNHM 99443: **D**); **F–K** *Hypselostoma lacrima* sp. n.: Holotype (HNHM 99444: **F–I**), Paratype (HNHM 99445: **J–K**). All images: B. Páll-Gergely.

#### Measurements

(in mm): SH = 1.14–1.34, SW = 1.22–1.36, AH = 0.43–0.5, AW = 0.49–0.53 (n = 10). See also Tables 9 and 10.

**Table 9.** Shell measurements (mm) for *Hypselostoma socialis* sp. n. from the type locality. For abbreviations see Table 1.

Specimen	SH	SW	AH	AW	SW/SH×100	AW/AH×100
holotype	1.34	1.36	0.46	0.51	101.49	110.87
paratype1	1.25	1.31	0.43	0.5	104.8	116.28
paratype4	1.22	1.28	0.46	0.5	104.92	108.7
paratype5	1.21	1.27	0.47	0.5	104.96	106.38
paratype6	1.22	1.26	0.45	0.49	103.28	108.89
paratype7	1.23	1.26	0.48	0.51	102.44	106.25
paratype8	1.18	1.22	0.45	0.49	103.39	108.89
paratype9	1.14	1.28	0.45	0.51	112.28	113.33
paratype10	1.26	1.31	0.5	0.53	103.97	106
paratype11	1.21	1.3	0.47	0.53	107.44	112.77

**Table 10.** Average, minimum value (min), maximum value (max), variance of values (var) and standard deviation of a set of values (stdev) for *Hypselostoma socialis* sp. n. (n = 10). For abbreviations see Table 1.

	SH	SW	AH	AW	SW/SH×100	AW/AH×100
Average	1.226	1.285	0.462	0.507	104.897	109.836
Min	1.14	1.22	0.43	0.49	101.49	106
Max	1.34	1.36	0.5	0.53	112.28	116.28
Var	0.0028	0.0014	0.0004	0.0002	9.3754	11.7788
stdev	0.0526	0.0378	0.0193	0.0142	3.0619	3.432

#### Differential diagnosis.

*Hypselostoma lacrima* sp. n. and *Hypselostoma socialis* sp. n. are the only species of *Hypselostoma* known from China. Some Chinese species formerly included in *Hypselostoma* have been reassigned to other genera (Yen 1939). *Hypselostoma dilatatum* Benthem Jutting 1962, *Hypselostoma rupestre* Benthem Jutting 1962 and *Hypselostoma annamiticum* Möllendorff, 1900 are approximately two times larger than *Hypselostoma lacrima* sp. n. and *Hypselostoma socialis* sp. n., and have more (5–8) apertural barriers. *Hypselostoma laidlawi* from Malaysia is similar in size to *Hypselostoma lacrima* sp. n. and *Hypselostoma socialis* sp. n., but it has a much narrower umbilicus and five apertural barriers.

*Hypselostoma lacrima* sp. n. has a much wider umbilicus than *Hypselostoma socialis* sp. n. Moreover, the spiral lines on the protoconch of *Hypselostoma socialis* sp. n. are weaker than those of the other species. The aperture of *Hypselostoma lacrima* sp. n. is heart-shaped with the sinulus vertically oriented, whereas the aperture of *Hypselostoma socialis* sp. n. is semi-quadrate and rounded with its sinulus positioned horizontally. The parietal lamella of *Hypselostoma socialis* sp. n. is interrupted and short (depressed Z-shaped), whereas that of *Hypselostoma lacrima* sp. n. is longer and straighter, lacking the conspicuous blade-like ridge visible in *Hypselostoma socialis* sp. n.

#### Etymology.

The name, socialis, (Latin: social) refers to the fact that this new species has been found together with three *Angustopila* species.

#### Type locality.

China, Guangxi (广西), Hechi Shi (河池市), Bama Xian (巴马县), cliffs at the southern edge of Jiaole Cun (交乐村), 590 m, 24°7.045'N, 107°7.847'E.

#### Distribution.

*Hypselostoma socialis* sp. n. is known from the type locality only (Figure 13).

#### Ecology.

As for *Angustopila fabella* sp. n.

#### Conservation status.

As for *Angustopila fabella* sp. n.

#### Remarks.

See under *Hypselostoma lacrima* sp. n.

### Genus Krobylos

Taxon classificationAnimaliaPulmonataHypselostomatidae

Panha & Burch, 1999

Krobylos Panha & Burch, Walkerana 10 (24): 127, 1999.

#### Type species.

*Krobylos pomjuk* Panha & Burch, 1999, by original designation.

### Krobylos sinensis

Taxon classificationAnimaliaPulmonataHypselostomatidae

Páll-Gergely & Hunyadi sp. n.

http://zoobank.org/A2630E1E-5259-4D3F-9C05-BB769B5EAFC3

Figures 9 , 10

#### Type material.

China, Guangxi (广西), Bose Shi (百色市), Leye Xian (乐业县), Chuandong Tiankeng Scenic Area (穿洞天坑景区), inner cliffs of the dolina, 1290 m, 24°48.430'N, 106° 29.277'E, leg. Hunyadi, A. & Szekeres, M., 09.09.2013., HNHM 99446 (holotype), HNHM 99447/1 (paratype), SMF 346522/1 paratype, HA/12 paratypes + 2 juvenile shells (not paratypes), PGB/1; China, Guangxi (广西), Hechi Shi (河池市), Tiane Xian (天峨县), Qimu Xiang (豈暮乡), cross towards Lahao Yan (拉号岩), 600 m, 24°51.130'N, 107°11.670'E, leg. Hunyadi, A. & Szekeres, M., 12.09.2013., HA/3 paratypes; China, Guangxi (广西), Hechi Shi (河池市), Huanjiang Xian (南丹县), cliffs above Dongning (峒宁) Village S of the Mulun Nature Reserve (木论国家级自然保护区), 530 m, 25°5.970'N, 107°57.639'E, leg. Hunyadi, A. & Szekeres, M., 17.09.2013., HA/3 paratypes.

#### Diagnosis.

A large *Krobylos* species with conical spire, rounded, regularly coiled whorls, large oval-shaped aperture, adnate parietal side and very weak indication of spiral striae on its dorsal surface.

#### Description.

Shell small, usually wider than high, only a single specimen from the Mulun Nature Reserve had the shell height and the shell diameter both measuring 2.7 mm; the 3.75–4.25 whorls are separated by a well-defined deep suture; whorls weakly angular, especially the penultimate whorl; protoconch light brownish purple, glossy, no notable sculpture visible; teleoconch light to dark purple, or pinkish, with blunt, irregularly course wrinkles; no spiral lines are visible under the microscope, but the SEM images revealed a hint of spiral striation on the lower half of each whorl (except for the last one); umbilicus open, narrow, (from ventral view), only its edge is covered by the peristome; aperture wide with its parietal part adnate to the penultimate whorl; peristome sharp, not thickened, not expanded nor reflexed; aperture reflected at columellar margin such that it covers the edge of the umbilicus.

**Figure 9.**
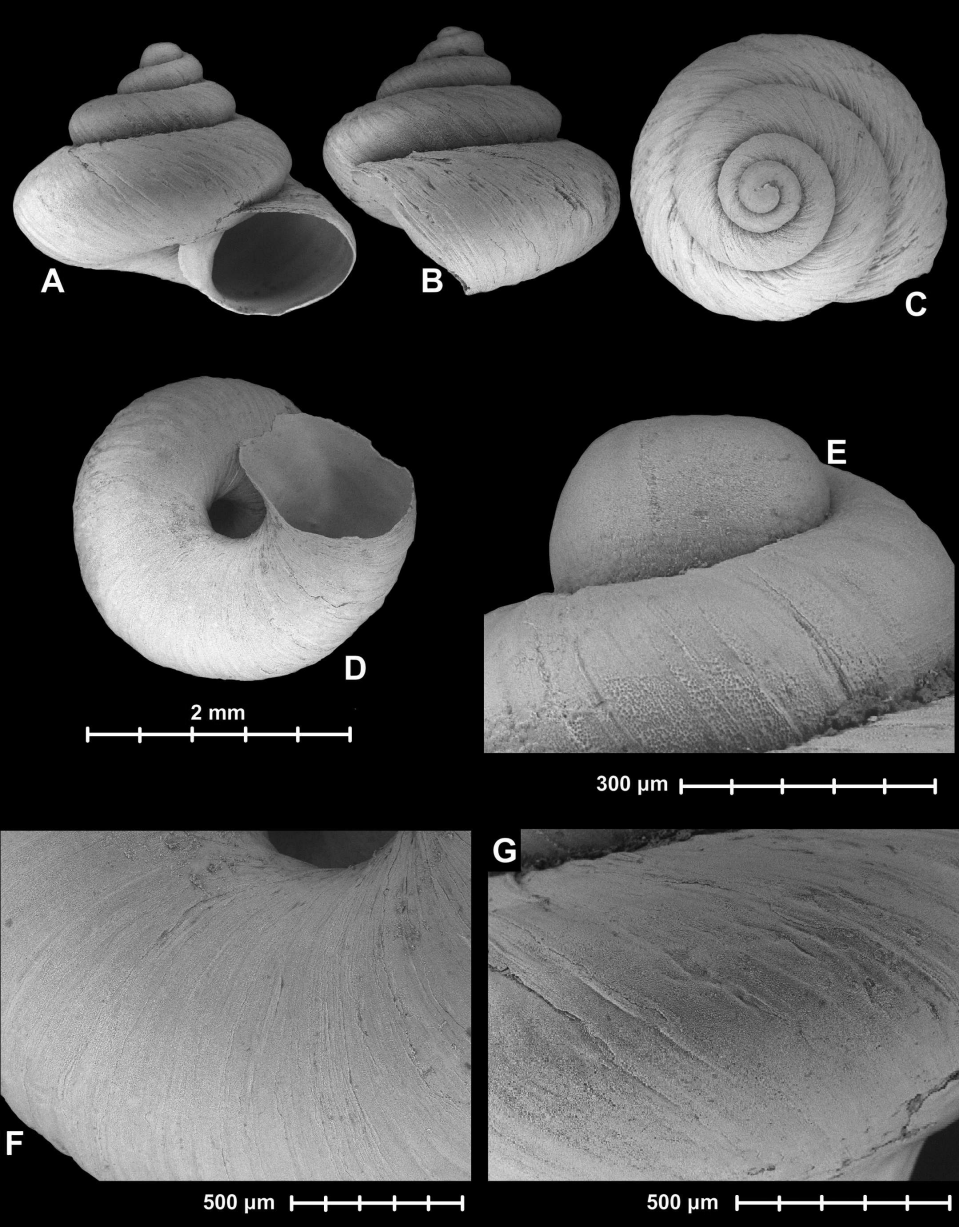
Holotype of *Krobylos sinensis* Páll-Gergely & Hunyadi, sp. n. (HNHM 99446). All images: B. Páll-Gergely.

**Figure 10.**
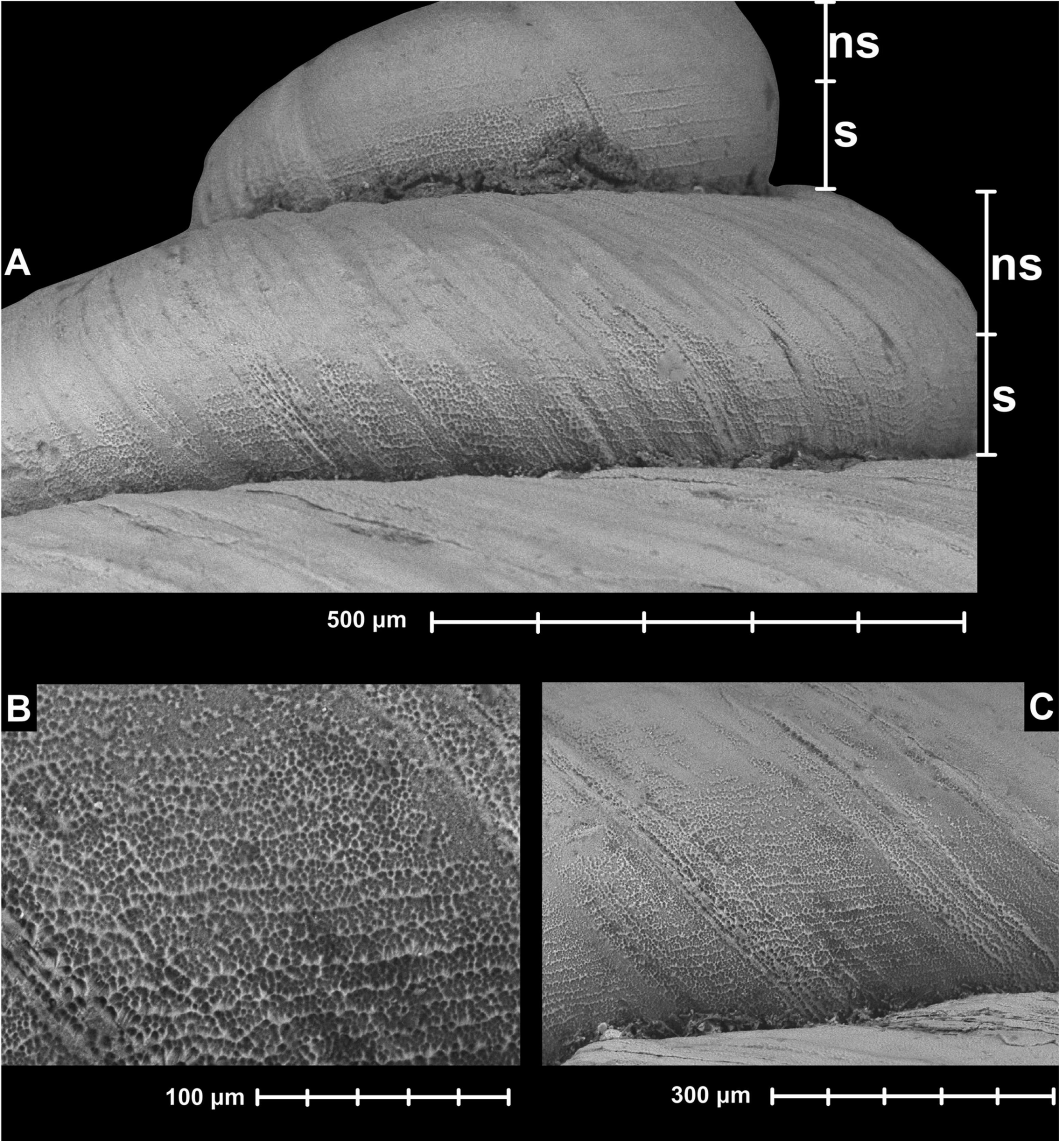
Sculpture of the holotype of *Krobylos sinensis* sp. n. (HNHM 99446). Abbreviations: NS: no spiral lines; S: spiral lines present. All images: B. Páll-Gergely.

#### Measurements

(in mm): SH = 2.2–2.7, SW = 2.5–3 (n = 13 from all populations).

#### Differential diagnosis.

*Krobylos sinensis* sp. n. differs from *Tonkinospira depressa* (Jaeckel 1950) by the larger size, rounded whorls and the absence of spiral sculpture on the upper sides of the whorls. The aperture of *Tonkinospira defixa* (Bavay & Dautzenberg, 1912) is not adnate, and its shell is much smaller than *Krobylos sinensis* sp. n. *Tonkinospira pulverea* (Bavay & Dautzenberg, 1909) has more rounded whorls and the entire surface is regularly spirally striated. *Tonkinospira pauperrima* (Bavay & Dautzenberg, 1909) has a much more elevated spire, narrower umbilicus and stronger spiral striae.

*Krobylos maehongsonensis* Panha & Burch, 1999 has a higher spire, a relatively larger aperture, sharper keel, weaker radial growth lines and more bulging whorls from dorsal view (in *Krobylos sinensis* sp. n. the whorls are ventrally more flat). *Krobylos kangkoy* Panha & Burch, 2004 (in Panha et al. 2004) has a much narrower umbilicus than the new species. *Krobylos pomjuk* Panha & Burch, 1999 also has a narrower umbilicus and a more depressed shell with a wider aperture. It is much smaller than *Krobylos sinensis* sp. n. Similarly as small, *Krobylos takensis* Panha & Burch, 2004 (in Panha et al. 2004) has a higher spire and more angled whorls. *Krobylos tampla* is even smaller bearing a narrower umbilicus. The aperture of *Krobylos veruwan* Panha & Burch, 2004 (in Panha et al. 2004) has a low palatal ridge, which is missing in *Krobylos sinensis* sp. n. Moreover, *Krobylos veruwan* is much smaller than *Krobylos sinensis* sp. n. and has a narrower umbilicus. *Pyramidula laosensis* Saurin 1953, which also likely also belongs to *Krobylos*, shows increased bulging whorls and a more pronounced closure of the umbilicus by the peristome.

#### Etymology.

The species is named after China, the country of its type locality.

#### Type locality.

China, Guangxi (广西), Bose Shi (百色市), Leye Xian (乐业县), Chuandong Tiankeng Scenic Area (穿洞天坑景区), inner cliffs of the dolina, 1290 m, 24°48.430'N, 106° 29.277'E.

#### Distribution.

*Krobylos sinensis* sp. n. has been found in three different localities in northern Guangxi Province (Figure 13). See also remarks on the distinctness of *Krobylos* and *Tonkinospira*.

#### Ecology.

Empty shells of this new species have been found in a soil sample at the base of large limestone rocks. It probably lives under stones and inside crevices.

#### Conservation status.

*Krobylos sinensis* sp. n. is reported from three sites in this study. This species may inhabit similar habitats in the same geographic area. At the moment, on a global scale, its distribution is likely limited to less than 5 sites, therefore these vulnerable narrow range endemics warrant conservation priority (Vu D2) in conjunction with the Guidelines for the IUCN Red List (IUCN Standards and Petitions Subcommittee 2014).

#### Remarks.

*Krobylos* was described as a group of toothless snails entirely lacking superficial microstructure (Panha and Burch 1999). *Tonkinospira*, on the other hand, has prominent spiral microsculpture over the entire surface. In this respect, *Krobylos sinensis* sp. n. is intermediate, because it has only very slight indication of spiral striae on the lower half of the whorls. This spiral sculpture is very faint or not visible under the microscope, but detectable using SEM images. We provisionally place *Krobylos sinensis* sp. n. in the genus *Krobylos* because of the very weak spiral striae. However, we remark that the distinctness of the genera *Krobylos* and *Tonkinospira* requires further study. *Krobylos sinensis* sp. n. is the only species assigned to *Krobylos* reported outside of Thailand. However, “*Pyramidula*” *laosensis* might also belong to the same genus.

## Discussion

**Figure 11.**
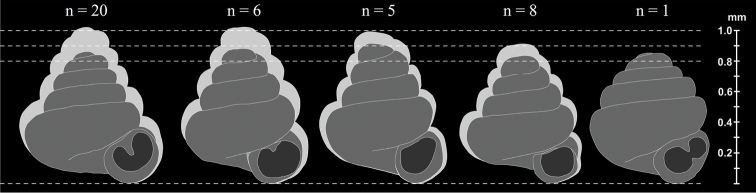
Comparison of the sizes of the five smallest *Angustopila* species. **A** *Angustopila fabella* sp. n. **B** *Angustopila szekeresi* sp. n. **C** *Angustopila elevata* **D** *Angustopila subelevata* sp. n. **E** *Angustopila dominikae* sp. n. Dark grey silhouettes represent the smallest, light grey the largest shells. The numbers above the shells indicate the number of shells measured.

Some of the new species reported in this study, especially the member of the genus *Angustopila*, have remarkably tiny shells. Adult individuals of *Angustopila subelevata* sp. n. (shell height = 0.83–0.91 mm, mean = 0.87 mm) and *Angustopila dominikae* sp. n. (shell height of the holotype = 0.86 mm) represent the smallest members of the genus *Angustopila*, since the smallest member of the genus so far was *Angustopila elevata* with 0.92–0.99 mm height (Thompson and Upatham 1997) (Figure 11).

**Figure 12.**
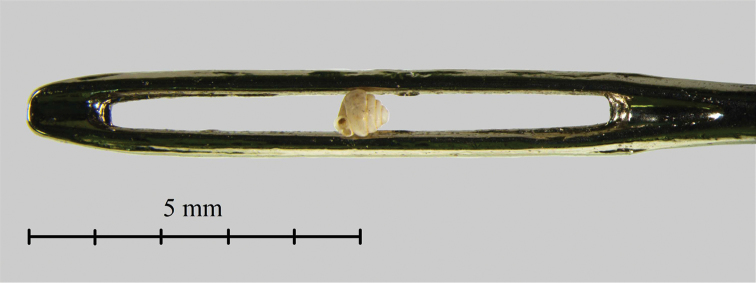
The holotype of *Angustopila dominikae* sp. n. in the eye of a sewing needle to picture its extraordinary small size. Photo: B. Páll-Gergely and N. Szpisjak.

**Figure 13.**
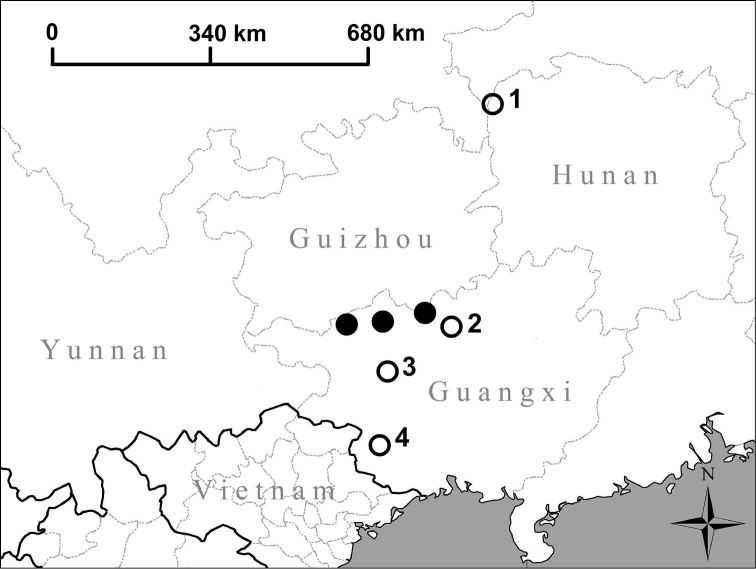
Map showing the distributions of newly described species of Chinese Hypselostomatidae. Filled circle: *Krobylos sinensis* Páll-Gergely & Hunyadi, sp. n. **1** Type locality of *Angustopila huoyani* **2** new locality of Angustopila cf. huoyani **3** Type locality of *Angustopila dominikae* Páll-Gergely & Hunyadi, sp. n., *Angustopila subelevata* Páll-Gergely & Hunyadi, sp. n., *Angustopila szekeresi* Páll-Gergely & Hunyadi, sp. n. and *Hypselostoma socialis* Páll-Gergely & Hunyadi, sp. n. **4** Type locality of *Angustopila fabella* Páll-Gergely & Hunyadi, sp. n. and *Hypselostoma lacrima* Páll-Gergely & Hunyadi, sp. n.

During a non-exhaustive literature survey (Powell 1979, Schileyko 1998a, 1998b, 2002, Panha and Burch 2005 for pulmonates; Boeters et al. 1989, Panha and Burch 2005, Liew et al. 2014 for operculate land snails), we found only very few reports of species smaller than 1 mm. The smallest land snail presented in these literature is “*Pupisoma* sp.” from Thailand, measuring “about 0.9 mm in length” (Panha and Burch 2005). Only a few genera containing species smaller than 1.5 mm according to Schileyko (1998a, 1998b, 2002), for example: *Pupisoma* (H = 1.3–3 mm; Schileyko 1998a), *Salpingoma* Haas 1937 (H = 1.3–1.5 mm; Schileyko 1998a), *Truncatellina* Lowe, 1852 (H = 1.2–2.5; Schileyko 1998b), *Acinolaemus* (H = 0.87–1.61, D = 0.65–1.92; Schileyko 1998b, page 255) and *Punctum* Morse, 1864 (D = 1–2 mm; Schileyko 2002). The height of 0.87 mm in *Acinolaemus* refers to a paratype of *Acinolaemus colpodon* Thompson & Upatham, 1997 measured from the base of the last whorl to the apex, but this is not the largest diameter of that shell. The largest measurement of that paratype is 1.05 mm from the base of the last whorl to the aperture. The diameter of 0.65 mm probably refers to the aperture height of *Acinolaemus rhamphodon* Thompson & Upatham 1997, which appears as a measurement of the shell width due to the shifting of data in the table presented in the original description (Thompson and Upatham 1997, page 227). *Paralaoma serratocostata* Webster, 1906, which is probably the smallest land snail in New Zealand, is generally less than 1.0 mm maximum shell dimension over a large part of its range (Powell 1979), but in some areas can reach 0.7 × 1.2 mm (Gary M. Barker, pers. comm.). As for operculated land snails, Liew et al. (2014) mentioned that the genus *Plectostoma* Adams, 1865 has a shell height of 1.0–3.7 mm. *Platyla minutissima* Boeters, Gittenberger & Subai, 1989, which is mentioned as the smallest European land snail, has a shell height of 1.1–1.25 mm. These data suggest that *Angustopila subelevata* sp. n. and *Angustopila dominikae* sp. n. are amongst the smallest land snails ever reported if the largest measurement of the shell is considered. If however, shell volume is calculated according to McCain and Nekola (2008) and Nekola (2014), there are even tinier land snails (e.g. Punctidae spp) occupying the lowest rung of the volume/size scale.

The smallest snails are, however, certainly marine species. The smallest recorded gastropod seems to be *Ammonicera minortalis* Rolán, 1992, ranging in size from 0.32 to 0.46 mm. Although a few marine species less than 1 mm are known, all of them are larger than *Ammonicera minortalis*. For example, Europe’s smallest gastropod, *Retrotortina fuscata* Chaster, 1896 measures 0.5–0.75 mm (Gofas and Warén 1998). Extremes in body size of organisms not only attract attention from the public, but also incite interest regarding their adaptation to their environment (Hanken and Wake 1993, Grebennikov 2008, Glaw et al. 2012). Investigating tiny-shelled land snails is important for assessing biodiversity and natural history as well as for establishing the foundation for studying the evolution of dwarfism in invertebrate animals. The present data are insufficient for addressing the evolutionary processes of miniaturization in land snails. However, we hope that these results provide the taxonomic groundwork for future studies concerning the evolution of dwarfism in invertebrates.

## Biogeography

The similarity between distantly distributed species (*Angustopila elevata* – *Angustopila subelevata*; *Angustopila tamlod* – *Angustopila huoyani*) and the two populations of *Angustopila huoyani* can be explained by three different hypotheses: (1) These populations may be connected with additional populations (i.e. via contiguous cave systems or interconnected river drainage basins) resulting in a continuous distributional area. The 500–1000 km gap between the known populations is therefore due to lack of additional exploration and thus, additional material; (2) they can be the results of rare long distance dispersal events; or (3) convergent evolution of shell traits. Our present knowledge is insufficient to reject any of these hypotheses.

## Supplementary Material

XML Treatment for Angustopila

XML Treatment for Angustopila dominikae

XML Treatment for Angustopila fabella

XML Treatment for Angustopila huoyani

XML Treatment for Angustopila subelevata

XML Treatment for Angustopila szekeresi

XML Treatment for Hypselostoma

XML Treatment for Hypselostoma lacrima

XML Treatment for Hypselostoma socialis

XML Treatment for Krobylos

XML Treatment for Krobylos sinensis
